# Visbrain: A Multi-Purpose GPU-Accelerated Open-Source Suite for Multimodal Brain Data Visualization

**DOI:** 10.3389/fninf.2019.00014

**Published:** 2019-03-22

**Authors:** Etienne Combrisson, Raphael Vallat, Christian O'Reilly, Mainak Jas, Annalisa Pascarella, Anne-lise Saive, Thomas Thiery, David Meunier, Dmitrii Altukhov, Tarek Lajnef, Perrine Ruby, Aymeric Guillot, Karim Jerbi

**Affiliations:** ^1^Computational and Cognitive Neuroscience Lab (CoCo Lab), Psychology Department, University of Montreal, Montreal, QC, Canada; ^2^Lyon Neuroscience Research Center, Brain Dynamics and Cognition team, INSERM UMRS 1028, CNRS UMR 5292, Université Claude Bernard Lyon 1, Université de Lyon, Lyon, France; ^3^Blue Brain Project, École Polytechnique Fédérale de Lausanne, Geneva, Switzerland; ^4^Institute for Applied Mathematics Mauro Picone, National Research Council, Rome, Italy; ^5^Aix Marseille Univ, CNRS, INT, Inst Neurosci Timone, Marseille, France; ^6^National Research University Higher School of Economics, Moscow, Russia; ^7^MEG Center, Moscow State University of Pedagogics and Education, Moscow, Russia; ^8^Center for Advanced Research in Sleep Medicine, Hôpital du Sacré-Coeur de Montréal, Montreal, QC, Canada; ^9^Inter-University Laboratory of Human Movement Biology, University of Lyon, University Claude Bernard Lyon 1, Villeurbanne, France; ^10^MEG Unit, University of Montreal, Montreal, QC, Canada

**Keywords:** visualization, neuroscience, python, open-source, brain, OpenGL, EEG, MEG

## Abstract

We present Visbrain, a Python open-source package that offers a comprehensive visualization suite for neuroimaging and electrophysiological brain data. Visbrain consists of two levels of abstraction: (1) objects which represent highly configurable neuro-oriented visual primitives (3D brain, sources connectivity, etc.) and (2) graphical user interfaces for higher level interactions. The object level offers flexible and modular tools to produce and automate the production of figures using an approach similar to that of Matplotlib with subplots. The second level visually connects these objects by controlling properties and interactions through graphical interfaces. The current release of Visbrain (version 0.4.2) contains 14 different objects and three responsive graphical user interfaces, built with PyQt: *Signal*, for the inspection of time-series and spectral properties, *Brain* for any type of visualization involving a 3D brain and *Sleep* for polysomnographic data visualization and sleep analysis. Each module has been developed in tight collaboration with end-users, i.e., primarily neuroscientists and domain experts, who bring their experience to make Visbrain as transparent as possible to the recording modalities (e.g., intracranial EEG, scalp-EEG, MEG, anatomical and functional MRI). Visbrain is developed on top of VisPy, a Python package providing high-performance 2D and 3D visualization by leveraging the computational power of the graphics card. Visbrain is available on Github and comes with a documentation, examples, and datasets (http://visbrain.org).

## Introduction

The aim of scientific visualization is to graphically illustrate datasets—which are can be highly complex- in order to provide a better understanding and facilitate the interpretation of the data. As scientific technologies continue to evolve, it becomes increasingly important to develop up-to-date and comprehensive visualization software capable of handling complex and large datasets. This is especially true in the field of neuroscience, which involves a myriad of neural recording types, and consequently, a wide and diverse range of possible data representations.

To date, Matlab (Mathworks, 2012) is one of the most widely-used commercial programming language for brain data analysis and visualization, thanks to a large number of toolboxes such as SPM (Penny et al., 2011), Brainstorm^1^ (Tadel et al., 2011), EEGlab^2^ (Delorme and Makeig, 2004) and Fieldtrip^3^ (Oostenveld et al., 2011). Alternative visualization solutions that run on non-commercial open-source programming environments, such as Python, are rare. These include high-quality packages such as MNE^4^ (Gramfort et al., 2013), PySurfer^5^, Nilearn^6^ (Abraham et al., 2014) or 3d slicer (Fedorov et al., 2012). Both MNE and Nilearn rely on Matplotlib for visualizations which is not suited for real-time interactions of brain imaging data involving thousands of data points. In addition, MNE also relies on PySurfer for 3D visualizations. PySurfer is built on top of Mayavi which contains a powerful rendering engine and allows smooth interactions. However, some issues have been reported when installing Mayavi, (which uses VTK), which may affect its user-friendliness.

In this context, we propose a Python open-source software called Visbrain, distributed under a Berkeley Software Distribution (BSD) license and dedicated to the visualization of neuroscientific data. Visbrain is built on top of VisPy (Campagnola et al., 2015), a high-performance visualization library that leverages the Graphics Processing Units (GPU). As a result, Visbrain efficiently handles the visualization of large and complex multi-dimensional datasets. The purpose of Visbrain is two-fold: (1) To provide within a common framework several Python-based visualization tools for neuroscientific data, (2) To allow users, including those with little or no programming skills access to high-end visualization functions, through a comprehensive documentation^7^ and a user-friendly API.

Many scenarii for the use of Visbrain are possible. For instance, a user with a set of intracranial EEG data could use visbrain to visualize in a first subplot the location of electrodes (e.g., NumPy array) either in individual or standard MNI space. Next, in a second subplot, the user may choose to project the data onto the cortical mesh (e.g., gamma power, *t*-values, decoding accuracies, etc). Additional subplots can be added, for example, to include data from other subjects, or various contrasts across experimental conditions. Because figures are dynamic, subplots can be added on the fly with various visualization objects such as connectivity, region of interest etc. The same procedure could be applied to M/EEG source data. Finally, each subplot can be animated and exported into a video file (e.g., animated GIF) or in a standard high-resolution publication-ready image file (e.g., PNG, JPG, TIFF).

With the release of this package and publication of this paper, we hope to develop a community of users that could facilitate extending and adapting this software to better cover the needs of researchers in neuroscience.

## Materials and Methods

The philosophy of Visbrain is to provide elementary visualization building blocks which can easily be combined in a modular manner, and to design a flexible and responsive graphical user interface (GUI) which can be used to change the active visualization parameters in real time. Visbrain is not designed to duplicate data analysis functions which are already available in well-established packages such as scipy^8^, pandas^9^, SciKits^10^, or statsmodels^11^, except when it serves illustration purposes.

### Programming Language and Code Guidelines

Although we initially considered Matlab and Julia (Bezanson et al., 2017) as language for Visbrain given their high level of abstraction, we finally chose Python since this mature and easy-to-learn language benefits from a large range of high-quality packages, a thriving and rapidly growing user community, and thorough documentation. Python software packages are portable, cross-platform, and easily distributed. More importantly, Python is free, open source, open access, and is thereby ideal for open science.

From a programming perspective, we paid particular attention to avoid memory-intensive data copy and to enable loading and processing of large dataset. Visbrain is hosted on GitHub^12^, and is documented using NumPyDoc, a Sphinx extension to generate NumPy-like documentation. We also provide illustrative examples and datasets. Code blocks are well-commented and follow PEP8 guidelines for code readability. Finally, package installation and features are tested under Linux and Windows through a continuous integration protocol (current coverage >85%).

### Dependencies

As Python 2.7 will not be maintained past 2020, Visbrain is a pure Python package for Python 3 only. Here is the list of Visbrain's dependencies are listed in Table 1.

**Table 1:** List of dependencies and package version.

**Package name**	**Purpose**	**Version**
NumPy	Scientific computing	≥1.13
SciPy	Scientific computing	-
PyQt5	Graphical user interfaces creation	-
VisPy	Graphics rendering	≥0.5.2
Matplotlib	Colors/colormaps related functions	≥1.5.5
Pillow	Screenshots and image file format support	-
PyOpenGL	Python binding to OpenGL	-

In addition to the above-mentioned packages, the use of some specific functionalities will require a few more dependencies. These include:

- Pandas (McKinney, 2011): for importing and exporting region of interest defined in the brain
- MNE-Python (Gramfort et al., 2013): alternative for loading sleep data files instead of using functions included in Visbrain
- Nibabel: for supporting certain file formats
- Tensorpac^13^ for computing phase-amplitude coupling
- Imageio: for Graphics Interchange Format (GIF) export

Finally, the Visbrain package can be downloaded using the python package manager pip^14^.

### GPU-Powered High-Speed Graphics

As the size, dimensionality and complexity of brain data continues to increase, data visualization tools have to be increasingly efficient, in particular if real-time interaction is needed. For example, high-density EEG or full-night sleep recordings can be associated with files of up to tens of gigabytes. Matplotlib, which is one of the most famous Python plotting libraries (Hunter, 2007), is primarily designed to provide static publication-quality figures and is unfortunately currently not suited for handling large data and user interactions. Seaborn^15^, which is built on top of Matplotlib is also not a viable option for the same reasons. Among libraries with mature development and real-time interaction, we also considered PyQtGraph^16^ and Glumpy^17^ (Rougier, 2015). Both options could certainly have been excellent alternatives. We rather considered the VisPy package (Campagnola et al., 2015), which is a high-performance interactive 2D/3D data visualization library leveraging the computational power of the GPU through OpenGL. The choice of VisPy was made mainly for the ease of installation and also because it is a combined effort by the authors of several visualization libraries (PyQtGraph, VisVis, Galry, and Glumpy)

The use of VisPy library is a critical component of Visbrain. By offloading most of the graphical rendering cost to the GPU, VisPy allows real-time interactivity, even for large datasets, while at the same time minimizing CPU overhead. As a result, Visbrain is able, on any modern-day laptop, to efficiently display large datasets and allows for real-time user interactions.

### Graphical Interface and User Interactions

Scientific visualization software often come with easy-to-use GUIs. Although most of the analyses can be performed in the command-line, such interfaces often greatly enhance the user experience. GUIs also allow users with no or little programming knowledge to use the software, making it more accessible to the scientific community.

To embed VisPy graphics in full-featured widgets, we chose to use the cross-platform C++ GUI toolkit Qt^18^, for which Python bindings are available (i.e., PyQt & PySide). Specifically, GUIs of the different Visbrain modules were built using the Qt designer tool and were then converted to Python code using PyQt.

### Documentation and Examples

Visbrain comes with a detailed step-by-step documentation built with Sphinx^19^ and hosted on the Visbrain website^20^. This documentation describes how to install Visbrain and use its modules. We also provide a description of GUI components and inputs for all class modules. Moreover, we provide a description of each graphical element using tooltips that appear when hovering corresponding widgets with the cursor. Lastly, we provide examples^21^ and python scripts that can be downloaded from the website. Some examples requires additional data to be fully functionals. Those data are either generated or comes from other open-sources softwares (i.e., MNE-Python, PySurfer, and Nilearn).

## Results

**Figure 1:**
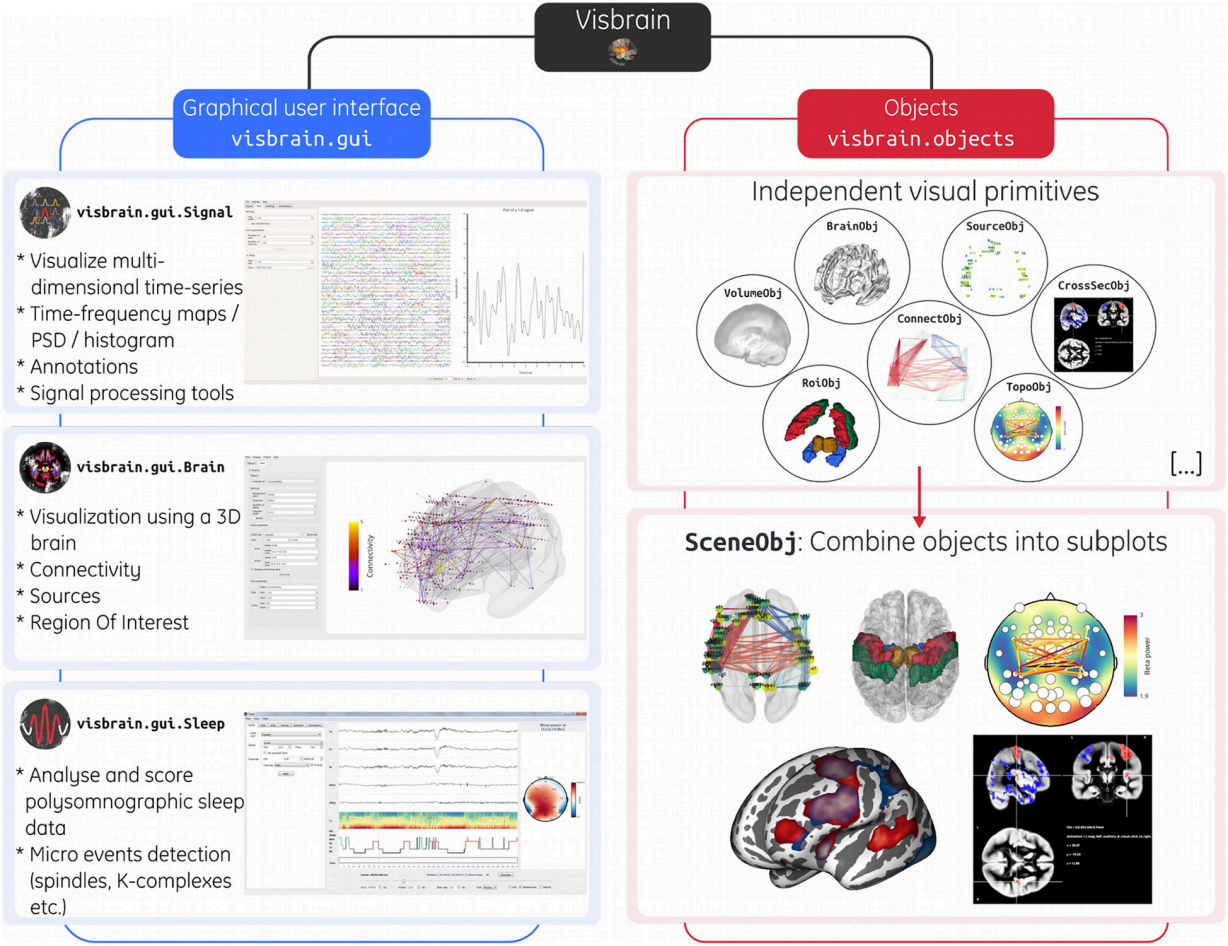
Architecture of the Visbrain software. The left branch in blue illustrates the three included graphical user interfaces (*Signal, Brain*, and *Sleep* and). For advanced users that want to interact programmatically with Visbrain, the right branch in red shows 6 of the 14 implemented objects in Visbrain. These objects are presented in circles to emphasize the fact that each of them is independent. Then, using the scene (*SceneObj*) these objects can be superimposed or juxtaposed into subplots inside a unique figure. The scene offers a finer grain control over the layout. Note that each subplot is interactive, meaning that rotation, translation and zoom can be applied in real time on each subplot.

From the user's perspective, Visbrain is subdivided into two main levels: *(1) Objects:* independent visual primitives that can be defined and used without the need for a GUI. *(2) Graphical user interface:* a user-friendly interface built on top of Visbrain objects for interactive visualization. The visbrain architecture is summarized in Figure 1.

### Objects

Objects represent the lowest level of Visbrain and can be considered as neuro-oriented visual primitives. Each object is highly configurable and serves a single visualization purpose. For example, the brain object (*BrainObj*) is used to display 3D brains. The definition of every object is independent, but some of them can interact together. For example, the activity of a source object can be projected onto the surface of the brain (see section Source object for the description of the projection). Those primitives bring modularity to Visbrain.

**Figure 2:**
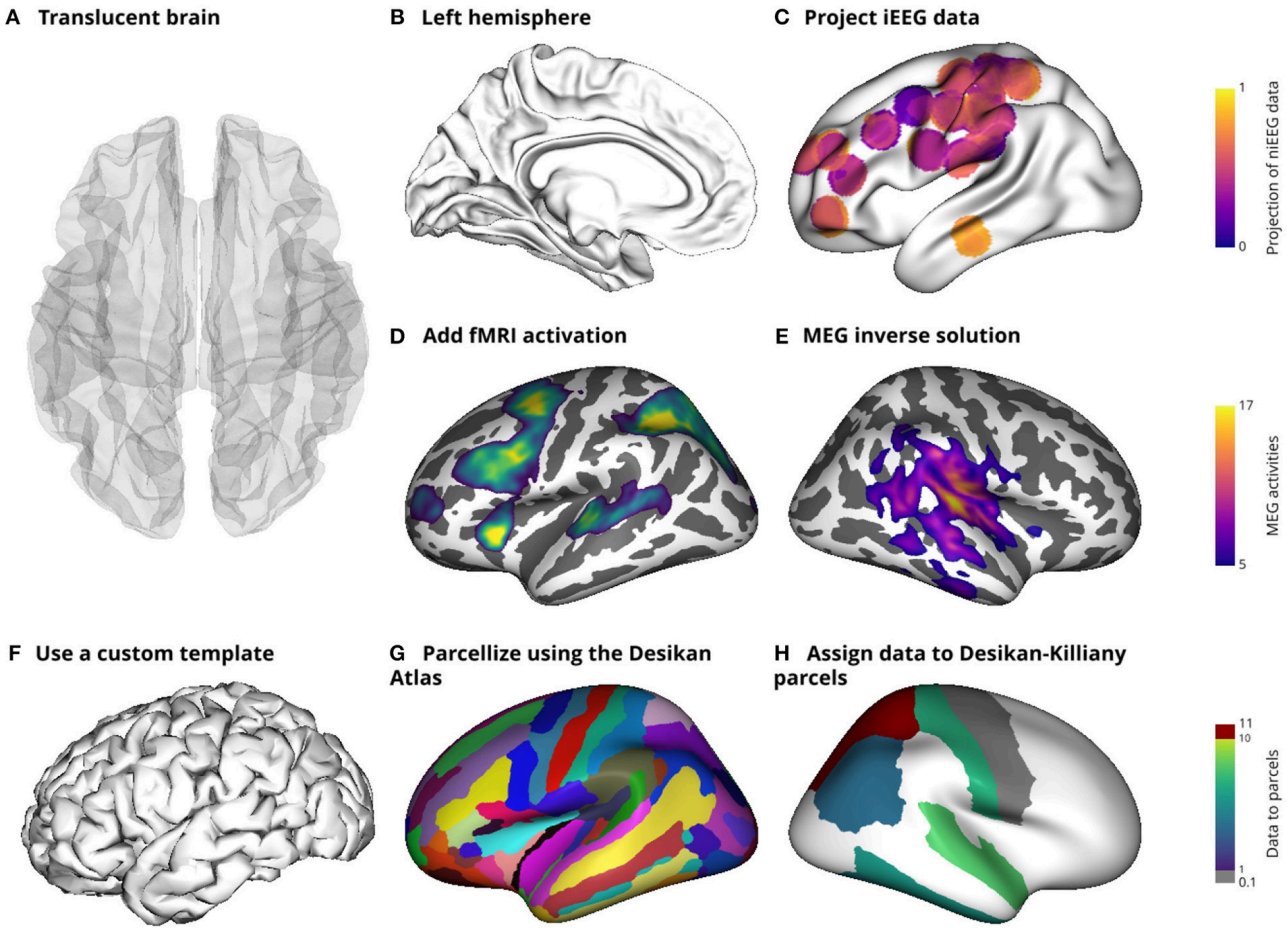
Illustration of the main features of the brain object (*BrainObj)*. This object delivers some basic features as the possibility to display a translucent or opaque brain mesh **(A)** or to pick only one hemisphere **(B)**. Intracranial data can also be projected onto the surface **(C)** and other recording modalities can also be displayed [fMRI activation **(D)** and MEG data **(E)**]. In addition, parcels can also be used **(G)** and data can be assigned to those parcels **(H)**. All of those subplots use MNI templates included with Visbrain, but the user can also define and save a custom template by defining subsets of vertices and faces **(F)**.

**Figure 3:**
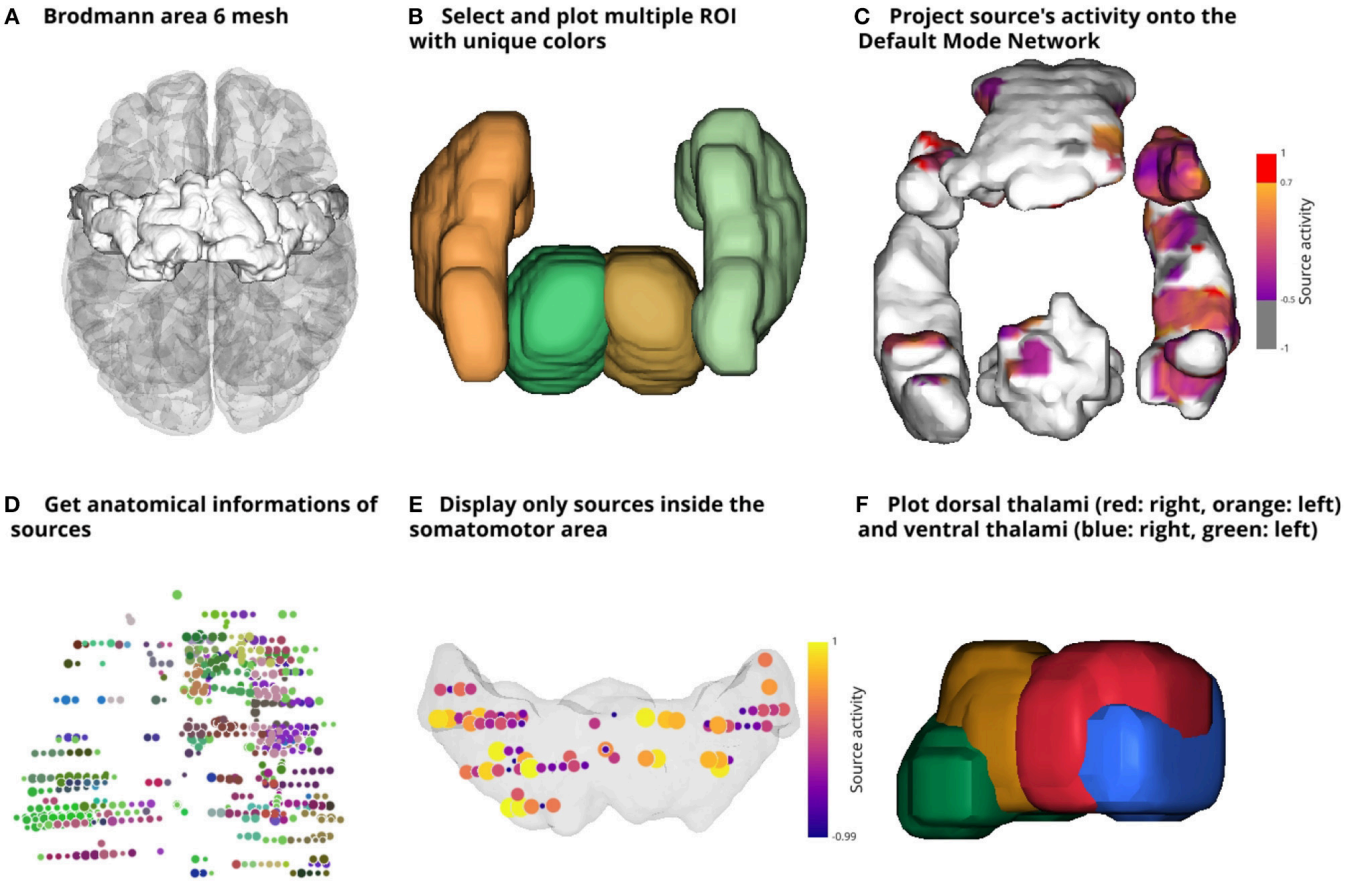
Illustration of the main features of the region of interest (ROI) object (*RoiObj*). Visbrain provides several default atlases that can be used to extract the mesh of specific regions **(A**,**B**,**F)**. In addition, the source object (*SourceObj*) can interact with the ROI object. For example, sources' activity can be projected onto the mesh **(C)**. The *RoiObj* can also be used to identify in which region a source is contained. Here, sources are color-coded according to the MIST **(D)** but a table with all of the anatomical informations can also be exported. Finally, it is also possible to keep only the sources that fall into the volume formed by the mesh **(E)**.

**Figure 4:**
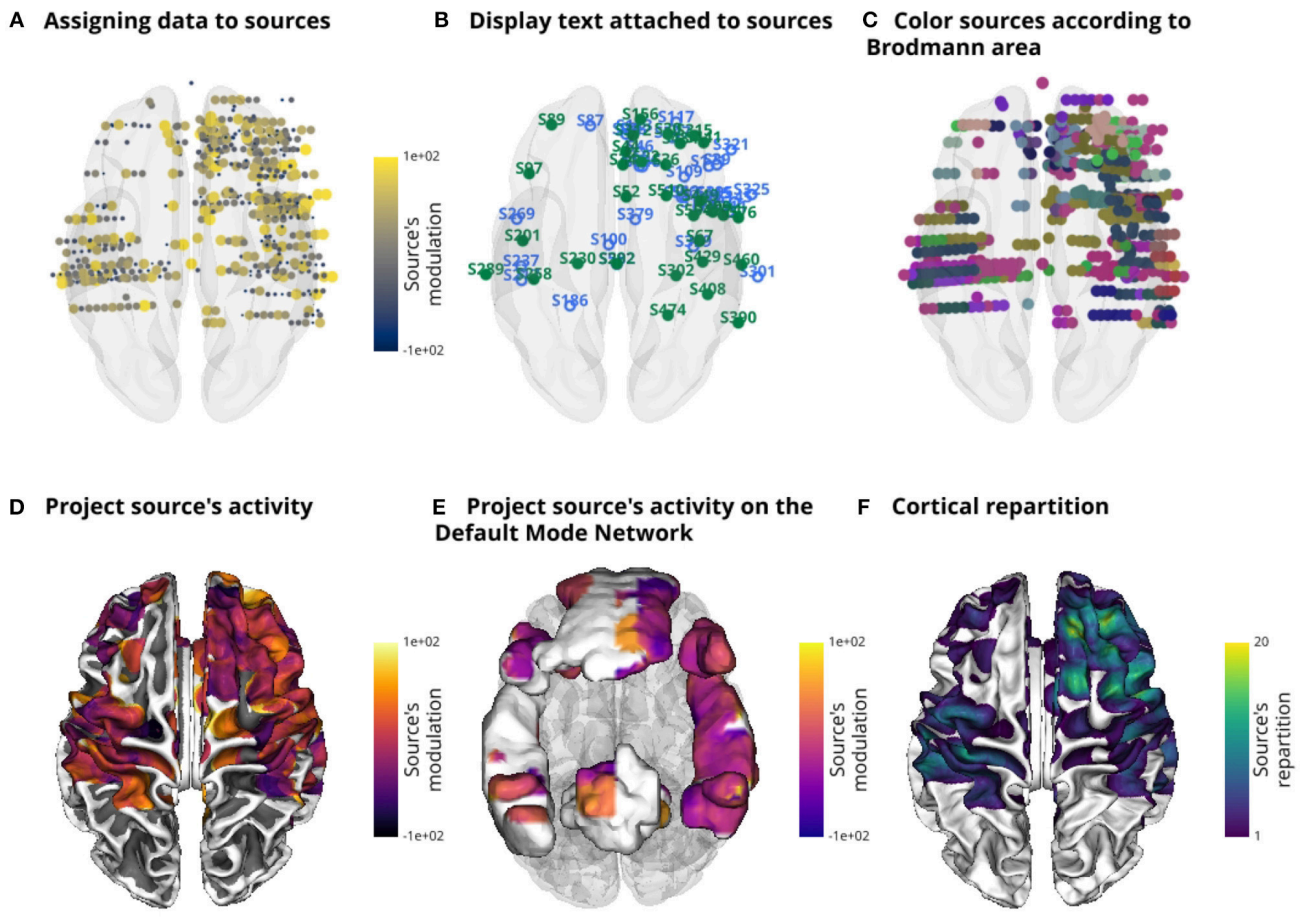
Illustration of the main features of the source object (*SourceObj*) using an intracranial dataset. Additional data can be assigned to sources and the color can either be individually defined or based on a colormap **(A)**. A text can also be attached to sources **(B)**. In a similar way to Figure 3D, here, sources are colored according to Brodmann areas **(C)**. The data attached to sources in **(A)** is then projected onto the surface of the brain **(D)** or onto the surface of the default mode network (DMN) **(E)**. Finally, the cortical repartition **(F)** is the number of contributing sources per vertex. It can be an interesting feature to estimate the number of sources that have contributed to each point of the cortical mesh when projecting source's data.

**Figure 5:**
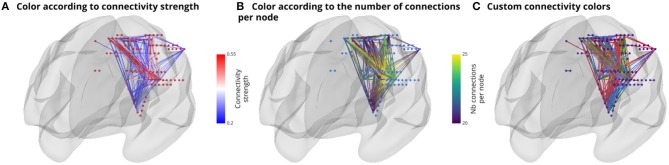
Illustration of the main features of the connectivity object (*ConnectObj*). The three sub-visuals express three coloring methods. The first method **(A)** is simply to color edges by connectivity strength. The second **(B)** color edges according to the number of connections per node and finally, **(C)** use colors that are manually defined.

**Figure 6:**
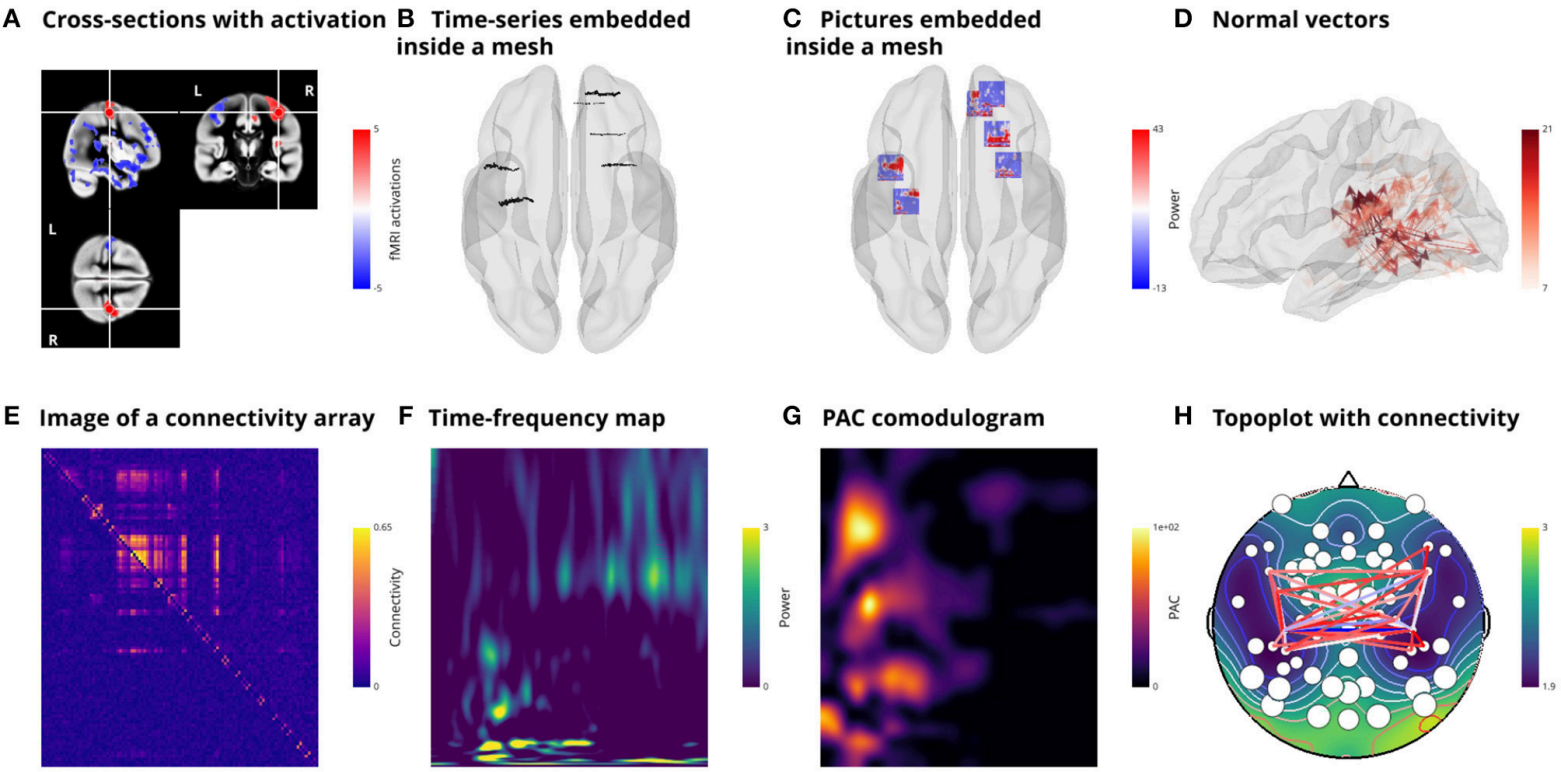
Illustration of additional implemented objects. **(A)** cross-section of fMRI data (*CrossSecObj*). The cross-section can be used to load background anatomical images and superimposed activations. It is also possible to move around the volume by clicking on it (still under development). Subplots **(B, C)** respectively illustrate time-series (*TimeSeries3DObj*) and pictures (*Picture3DObj*) embedded inside the mesh. Here, the pictures are time-frequency maps. **(D)** Plot vector-valued (*VectorObj*) MEG inverse solution. Visbrain also contains objects to plot images (*ImageObj*) as illustrated in **(E)** with a connectivity matrix, time-frequency maps (*TimeFrequencyObj*) **(F)**, phase-amplitude coupling (*PacmapObj*) **(G)**. Finally, the *TopoObj* can be used to plot topographic representations of EEG data, draw levels and connectivity links between EEG sensors **(H)**.

Those objects can then be superimposed and juxtaposed inside subplot (see section Scene object). It should be noted that Figures 2**–6**, that combine these objects, were not post-paginated (i.e., static rendering), but were generated from the scene object as real-time interactive figures. Finally, objects can also be animated, either independently or within subplots. Furthermore, such animations can be exported as a gif file.

#### Implemented Objects

The current version of Visbrain implement many classes, among them 14 defines visual objects that can be directly imported from *visbrain.objects* and be added to a scene. The API for interacting with those primitives are described inside the documentation^22^ (see Table 2 for a list of the visual objects).

**Table 2:** List of the 14 visual objects implemented in Visbrain.

**Object name**	**Description**
*BrainObj*	3D brain with vertices colored according to data
*ColorbarObj*	Colorbar associated with another object
*ConnectObj*	3D connectivity lines between nodes
*CrossSecObj*	Interactive fMRI cross-section (axial, sagittal and coronal views)
*HypnogramObj*	Hypnogram for sleep data
*ImageObj*	Images or 2D arrays
*PacmapObj*	Phase-amplitude coupling of a single time-series (PAC)
*Picture3DObj*	Images distributed in 3D space
*RoiObj*	Volumetric region of interest
*SourceObj*	Sources distributed in 3D space (intracranial/MEG/EEG)
*TimeFrequencyObj*	Time-frequency map of a single time-series
*TimeSeries3DObj*	Time-series distributed in 3D space
*VectorObj*	3D vectors
*VolumeObj*	Volumetric data

#### Illustrations of the Main Functionalities of the Objects

In this section, we provide a non-exhaustive review of the main features of some of the most used objects.

##### Scene object

Probably one of the most useful objects of Visbrain is called the scene (*SceneObj*). The scene is not a visual primitive in the sense that it cannot be used to represent any kind of brain data. Instead, it is an equivalent of Matplotlib's subplots meaning that objects can be superimposed inside sub visuals or displayed side by side. While requiring from the user some modest programming skills, the scene presents three major advantages: 1) it is undoubtedly a more flexible way to meet some specific visualization needs, 2) scenes can be integrated inside loops, on a local computer or on a distant server which means that the production of figures can easily be automated,3) the layout of figures for scientific publications can be assessed using this scene and 4) subplots remains interactives which allow the user to continue to interact with each object independently. Figures 2**–6** are defined using the *SceneObj* and the code snippet 1 illustrates a basic example of how to use the scene object to define the layout of a figure.

**Code Snippet 1:**
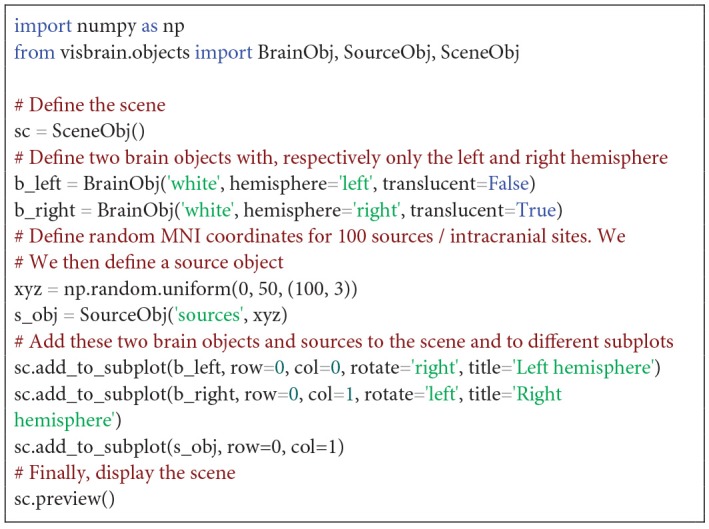
Display the left and right hemispheres into two separate subplots along with random MNI sources/contacts/electrodes.

##### Brain object

The brain object (*BrainObj*) can be used for every scenario where a 3D brain mesh is needed. Left and right hemispheres can be individually displayed on a translucent or opaque mesh. In addition, overlays of data can also be added to the mesh to illustrate fMRI, M/EEG or intracranial activations. The brain object capabilities are summarized in Figure 2.

##### Region of interest object

Regions of interest (ROI) are labeled volumes, i.e. a 3D array of voxels associated with an anatomical label (e.g., “Somatosensory cortex”). By default, Visbrain supports Brodmann areas, the Automated Anatomical Labeling (AAL; Tzourio-Mazoyer et al., 2002), the Talairach atlas (Talairach and Tournoux, 1993) and the Multiresolution Intrinsic Segmentation Template (MIST; Urchs et al., 2017). New ROIs can also be defined by providing a 3D array for the volume and labels. The *RoiObj* provide the users with an interface to the volume and let them extract the mesh of a specific region and assign different colors to it. The code snippet 2 shows how to extract the mesh of the thalamus and Figure 3 demonstrates some core features of this object.

**Code Snippet 2:**
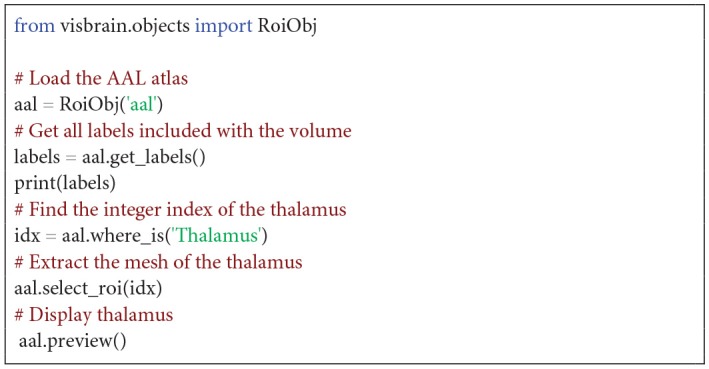
Display the left and right thalamus

##### Source object

The source object (*SourceObj*), depending on the recording modality can either represent intracranial recording sites, M/EEG sensors or reconstructed source activity. A text and marker color can also be assigned to each source. In addition, data can be provided to those sources to have marker radius proportional to the data.

Another useful and relatively rare feature among existing software is the ability to use the source object to project intracranial data onto a mesh (e.g the cortical surface of the brain or onto ROI). Usually, the implantation of intracranial electrodes is subject dependent, which then poses a problem to visualize the results across subjects. Cortical projections can solve this limitation and have been previously used (Combrisson et al., 2017a). When projecting the data, each vertex in the mesh can be considered a bin which simply accumulates the data (e.g. beta power) from nearby intracranial sites. The data from all sites that are under a certain radius (10 mm by default) contribute to this bin. It is what gives the circular aspect to this projection (see Figure 2C). Instead of projecting data, it is also possible to project the number of sources that contribute to each point of the mesh. In this case, the color indicates how many sources participated. Finally, the last feature that we want to point out is the possibility to get anatomical informations on sources using the ROI object. For example, this can be used to deduce in which Brodmann area a source (e.g., or an electrode) is contained. Those functionalities are presented in Figure 4.

##### Connectivity object

The connectivity object (*ConnectObj*) is used to draw connectivity lines between nodes. We provide three coloring methods: 1) set color to the edges according to connectivity strength, 2) set color to the node according to the number of connections per node or 3) set color of edges manually. Figure 5 shows an example of those differences in coloring methods. Display of directional connectivity is still an experimental feature and therefore is not presented.

##### Other objects

Visbrain contains several other objects serving various purposes, such as drawing vectors, displaying images, time-frequency maps, and phase-amplitude coupling comodulograms. For EEG recordings, topographic representations such as cross-sections previously shown for anatomical and functional MRI can also be plotted. Figure 6 summarizes the use of these objects.

For a list of all supported data types for the various objects we refer the reader to the online API documentation^23^

### GUI Based Interfaces

The main objective of GUIs is to connect and centralize the main features of the smaller visualization bricks. At the moment, Visbrain contains three interfaces:

- ***Signal*:** for the inspection of time-series and spectral properties (PSD power and time-frequency map decomposition, phase-amplitude coupling,…)
- ***Brain*:** for any type of visualization involving a representation containing an opaque or translucent brain
- ***Sleep*:** for plotting, staging, and analyzing sleep data

GUI can be imported from *visbrain.gui*. Those interfaces share the following properties and functionalities:

- A responsive GUI with a common graphical design and structure: a “quick settings” panel disposed on the left (which can be hidden or displayed) and plot on canvases displayed on the rest of the screen. This settings panel contains PyQt widgets to control objects' properties and apply changes in real time.
- The use of VisPy to exploit GPU capabilities.
- A “File” menu to import and export files (such as datasets, annotations, …). From this menu, it is also possible to save and load the GUI state, i.e., the value of each PyQt graphical elements (checkboxes, comboboxes,…). The configuration is saved into a text file with a JavaScript Object Notation (JSON) structure and can later be reloaded to retrieve the session.
- A “Display” menu that controls which elements are displayed or hidden on the screen.
- A “Help” menu to open an informative web page in a browser about the current module and features. This help can also be downloaded in PDF format.
- The support of keyboard shortcuts and mouse events (left and right clicks, double clicks, mouse wheel scrolling,…). The list of supported shortcuts is referenced in a table accessible from the help menu.
- A screenshot window to either export the entire window or select canvas with controllable size, resolution, and printing options. Visbrain supports several standard picture formats (such as PNG, JPG, PDF, EPS, or TIFF). The transparency and background color can also be controlled from this window. An “auto-crop” option can also be checked to automatically crop the exported image to the closest non-background pixel.

#### Signal: Time-Series Visualization and Spectral Properties

**Figure 7:**
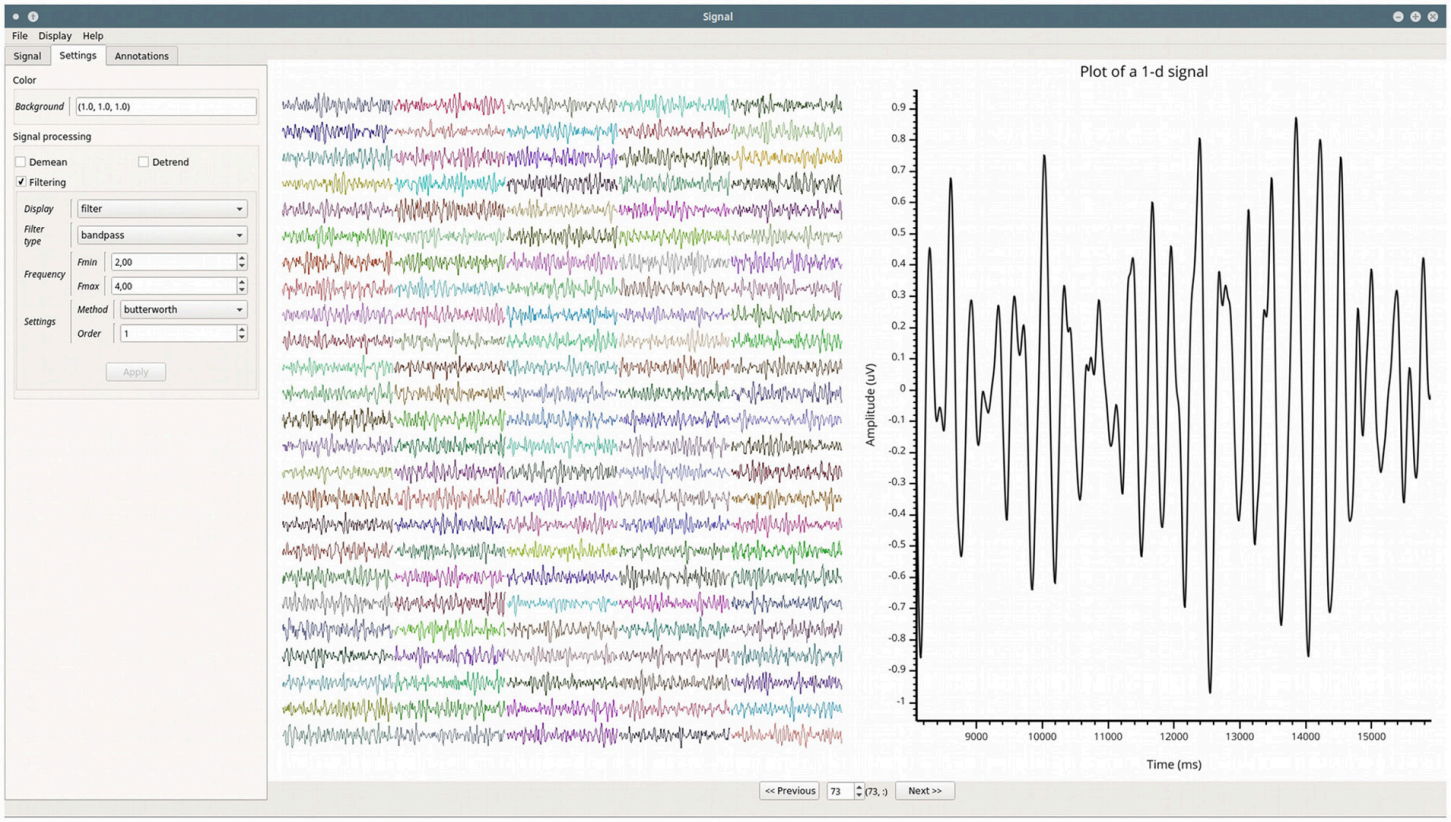
Example of the GUI of the *Signal* module. Leftmost is the setting panel, and side-by-side are all of the time-series re-arranged into a 2D clickable grid and rightmost, an enlarged version of one of those time-series.

A common first step when exploring electrophysiological data consists of inspecting time-series. This inspection phase is useful to get an idea of the shape of the signals, as well as quickly detecting artifacts, epileptic spikes, eye movement contamination, etc. Spectral properties such as power spectrum density (PSD) or time-frequency maps are complementary tools for such quality control and data exploration. Such data inspection can be complicated for multi-dimensional datasets (e.g., number of trials x tasks x time points). To address this issue, we developed the *Signal*^24^ module for the visualization of multidimensional signals. The GUI is divided into two layouts presented in Figure 7. On the left, the dataset overview. This consists of a grid where all of the time-series in the dataset are displayed. Multi-dimensional arrays are systematically reshaped into a 2D grid. On the right, the detailed view of a single signal. This second layout can be used to plot the time-series, the PSD or the time-frequency map.

##### Grid disposition

The usefulness of this data exploration module is demonstrated by one of the VisPy examples^25^, in which thousands of signals, each having thousands of points, can be instantly plotted using the GPU graphics rendering. These signals are presented in a two-dimensional grid and the user can zoom on each of them. Since this grid of signals can be useful for plotting electrophysiological data, this representation has been integrated into the *Signal* module (see Figure 7). The aim of this grid is to provide an overview of the entire dataset in a convenient way to visualize all the time-series at once. In order to take advantage of the width and height of the screen, the program tries to determine an optimal number of rows and columns for the grid. A title can also be added on top of each signal of the grid to facilitate the orientation of the user. To better visualize the signal on a specific channel, the user can double-click on it in the grid. This enlarges the selected signal by opening it in the second layout.

##### Plotting forms

**Figure 8:**
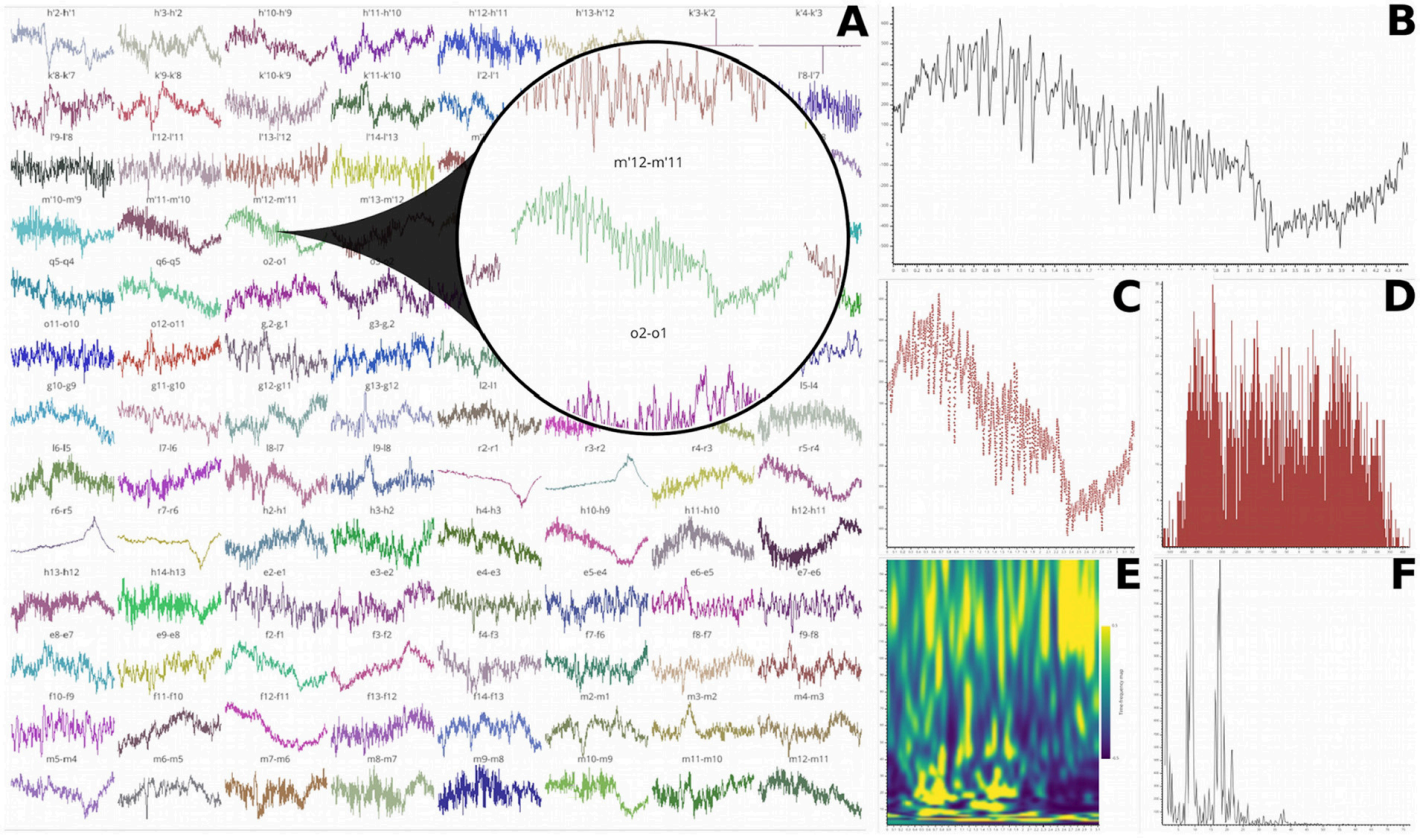
Plotting capabilities of *Signal*. **(A)** 104 intracranial recording sites of 4,000 time points each are rearranged into a clickable 13 rows by 8 columns grid. A double-click on one signal of the grid enlarges it in the second layout. This enlarged time-series can either be displayed as a continuous line **(B)** or a cloud of points **(C)**. A histogram can also be computed **(D)** as well as the time-frequency map using Morlet's wavelets **(E)** or the power spectrum density **(F)**.

In addition to the grid, a second layout is provided to inspect one time series at a time. The default plotting method is a continuous line but it can be changed to markers for a cloud of points. We also included the possibility to compute the histogram, the PSD or a highly configurable wavelet-based time-frequency map (such as normalization method, baseline bounds, etc.). The grid and those plotting forms are summarized in Figure 8.

##### Annotations, thresholding, and signal processing tools

**Figure 9:**
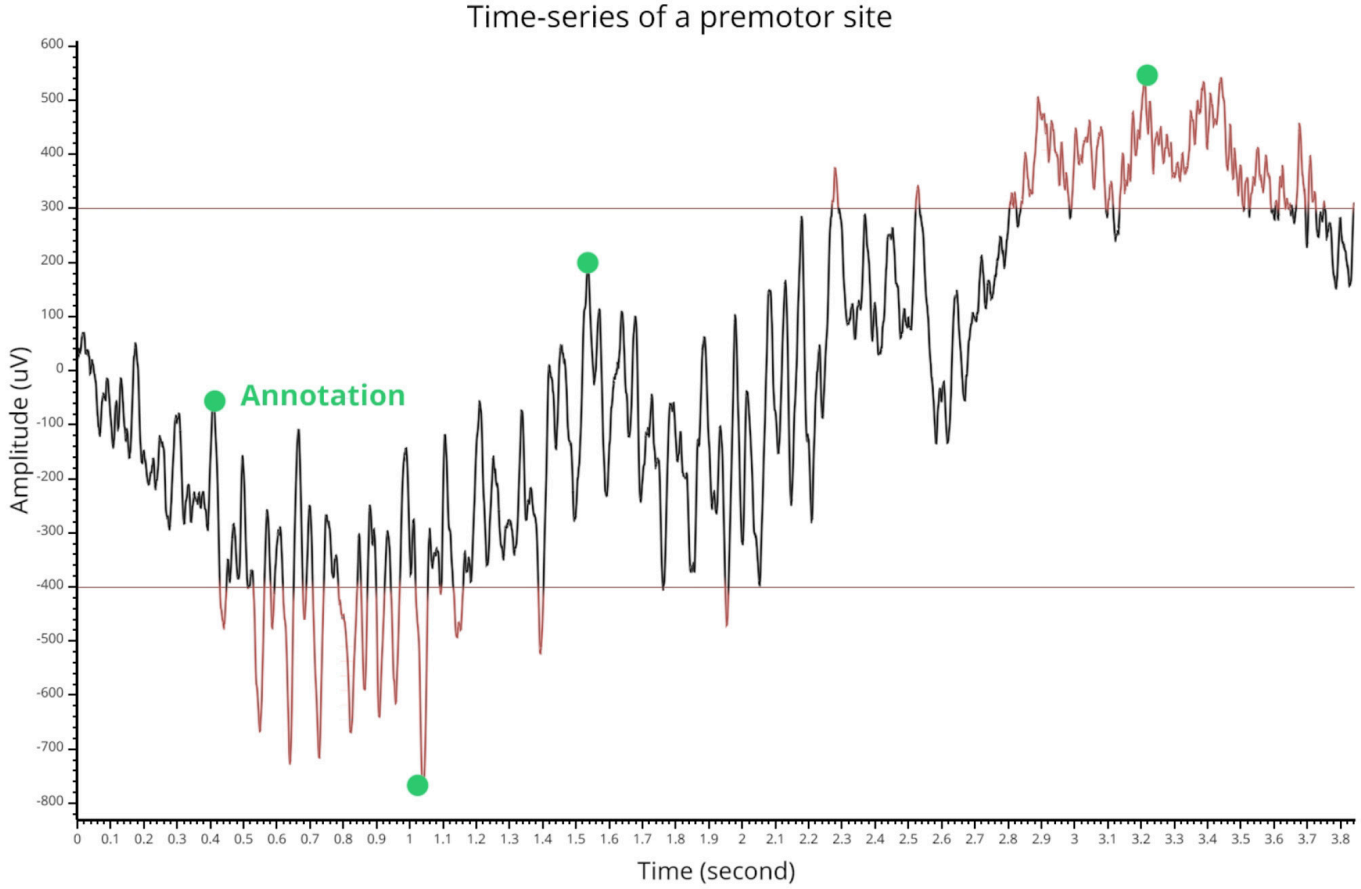
Thresholding and annotation example of an intracranial time-series. The two horizontal lines indicate the threshold values and time points that are either above or under are turned in red. The green markers show inserted annotations that can also be exported.

This module also supports annotations by double-clicking on the canvas that contains the single time-series. All inserted annotations are referenced in a table that can be exported or imported. Selecting a row of this table displays the annotated trial with associated annotations. Then, the *Signal* module also allows the user to define a lower and upper threshold for the identification of time-series extrema. These annotations and thresholding capabilities are summarized in Figure 9. We also included some signal processing tools such as filtering, detrending, smoothing, and demeaning.

#### Brain: Visualization on a 3D Brain

*Brain*^26^ is the second graphical interface that has been developed for all types of visualizations involving a 3D brain. This interface is not intended to provide extra functionality compared to what can be done with the Visbrain objects and scenes. Instead, it provides a GUI to control these objects and the interactions between them.

##### Object and colorbar control

**Figure 10:**
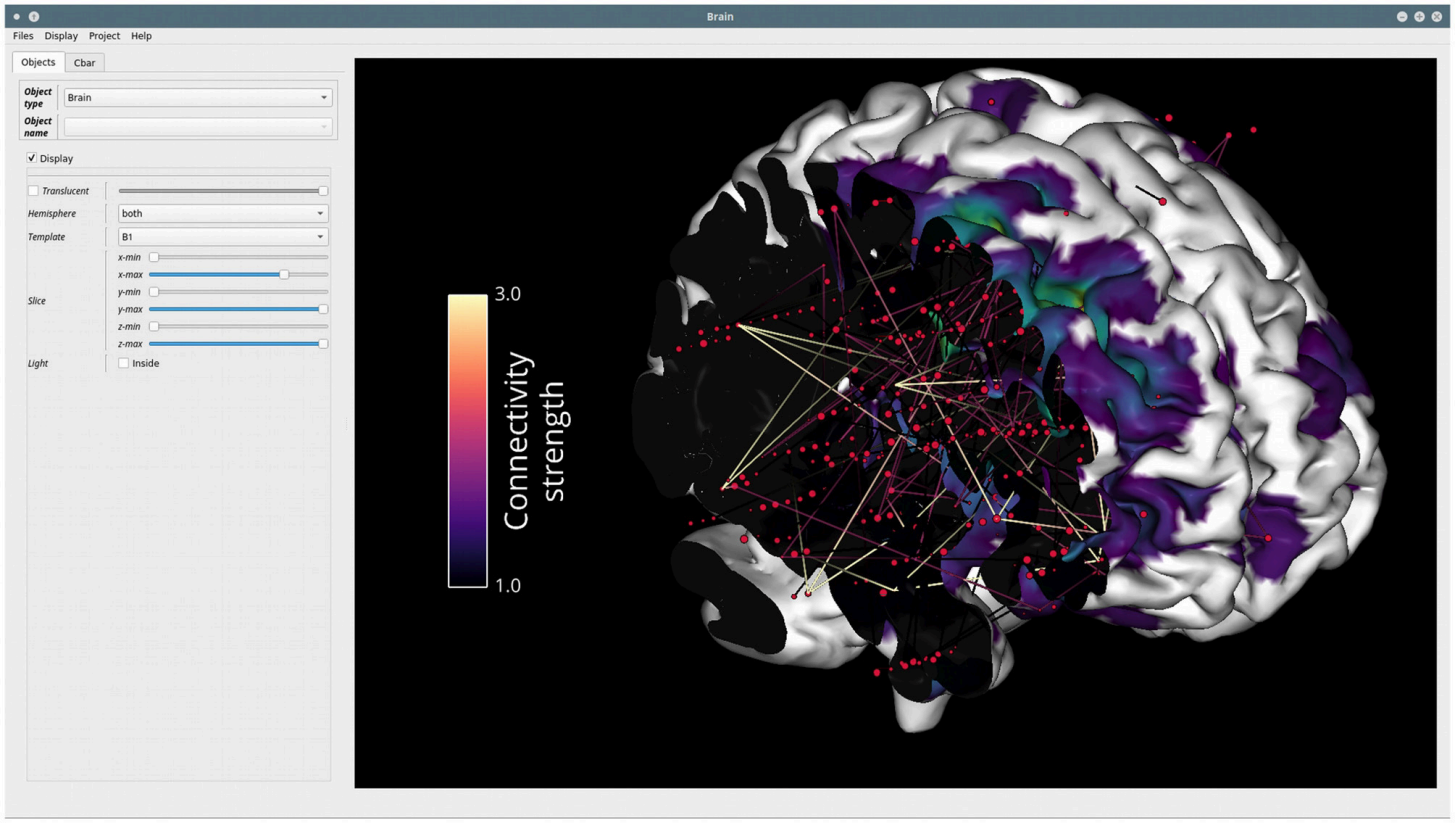
Example of the GUI of the *Brain* module. The settings panel on the left can either be displayed or hidden. This panel contains two tabs: *Objects*, in order to control the properties of each visual class (e.g., *BrainObj, SourceObj*, etc.) passed to the interface and *Cbar* for controlling the colorbar and color properties of a selected object. On the right, the main canvas contains the MNI brain with sources and connectivity links between those sources. This canvas allows fluid rotation, zoom and translation, but also mesh slicing along the (x, y, z) axes. Here, the colorbar of connectivity strength is displayed but it can also be hidden.

**Figure 11:**
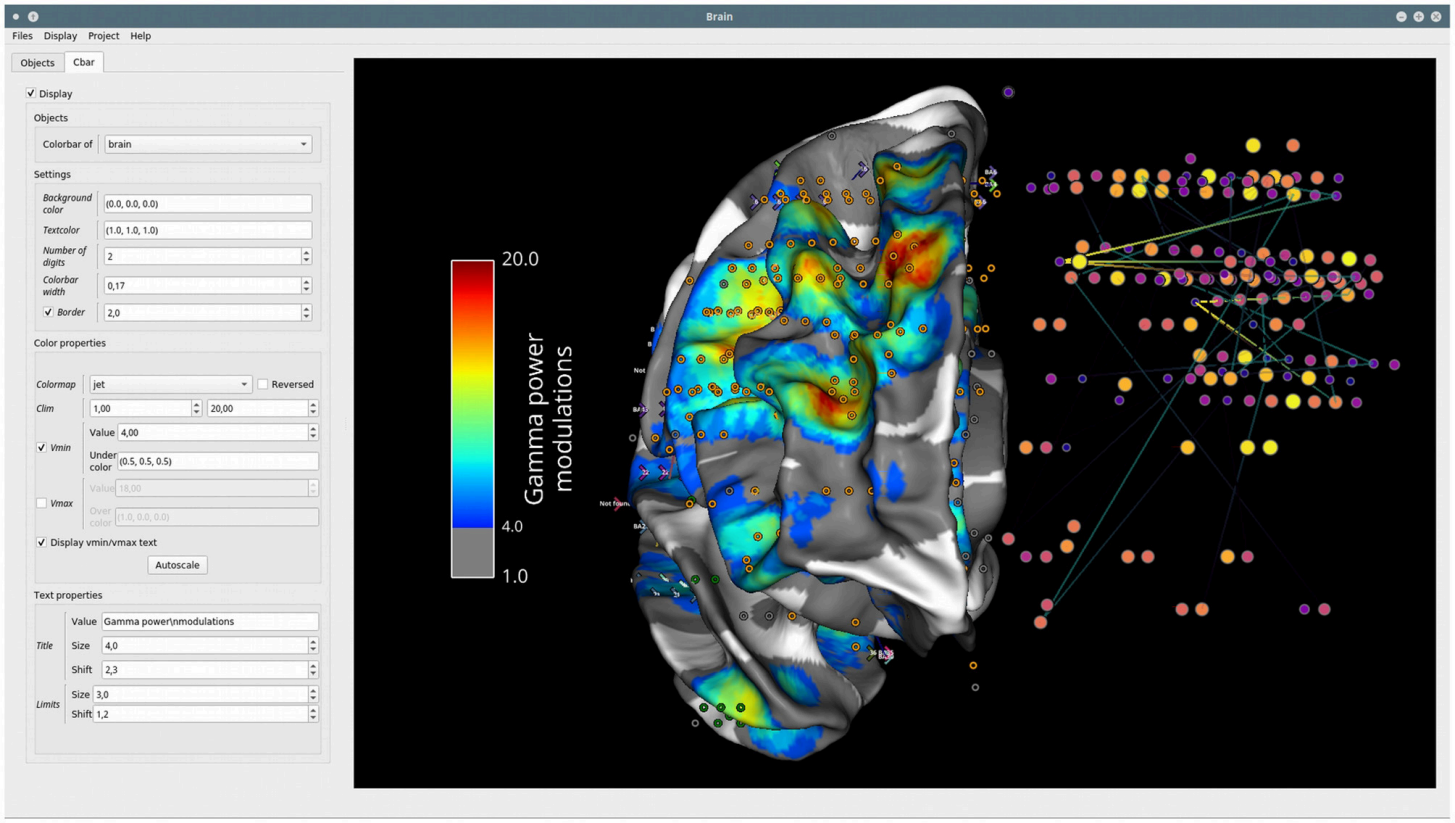
Colorbar control. The *Cbar* tab of the settings panel contains all of the properties to design the colorbar of a specific object (width, colormap, limits, lower and upper thresholds, title, etc.). In addition, these properties can be modified for each object. Here, the widget controls the colorbar for the data projected onto the surface of the brain.

The *Brain* class can take as input objects or list of objects from the following classes: brain, sources and connectivity (*BrainObj, SourceObj* and *ConnectObj*), 3D time-series, pictures, and vectors (*TimeSeries3DObj, Picture3DObj, VectorObj*) and volume related objects (*VolumeObj, CrossSecObj*, and *RoiObj*). Any object passed to the *Brain* class can then be directly controlled from the *Object* tab inside the settings panel (see Figure 10). In addition, since each visual object has its own color properties, the colorbar can be controlled individually for each of them from the *Cbar* tab (see Figure 11).

##### Class method for command line interaction

All the functionality and object properties that are accessible from the GUI can also be used and set using *Brain* class methods. The use of methods does not require the graphical interface to be open, even for screenshots. Hence, users can leverage those class methods in custom python scripts to speed up the production of large sets of figures. All of these methods are referenced in the documentation^26^.

#### Sleep: Polysomnographic Data Visualization and Edition

*Sleep*^27^ is the Visbrain module dedicated to the visualization and analysis of sleep data and has been previously described (Combrisson et al., 2017b). It should be noted that new features have been added to the *Sleep* module since the publication of this article, such as the possibility to replace the default event detections with custom external algorithms. This allows different sleep research teams to use the same data visualization platform while still keeping their custom, lab-specific, algorithms for the detection of transient events during sleep.

### API and Scripting

As visbrain is subdivided into two main levels (Objects and GUI where GUIs are built on top of objects) we also provide an API for higher level interactions. GUIs are of course ideal when user interaction is needed. That said, GUIs are obviously not intended to be embedded inside loops for scripting. Conversely, the object level offers less options for graphical interactions (except for translations and rotations) but is ideal for scripting, automating and streamlining the production of high-quality figures. This could be implemented either on a local computer or remotely on a distant server. In addition, the API provision also implies that other toolboxes that have intensive visualization needs (e.g. MNE-Python) can benefit from this API and the modularity of Visbrain objects. The full Visbrain's API can be found in the online documentation^28^.

## Discussion

### Summary

The ever-growing complexity of neuroimaging recording techniques, relying on analyses in higher-dimensional space and on larger datasets, are gradually transforming brain data visualization into a real challenge for the existing body of neuroimaging software. This challenge is further complicated by the difficulty of meeting the specific needs from individual research teams and by license compatibility issues with proprietary software. With these problems in mind, we propose Visbrain, a versatile Python 3 package for multi-modal brain data visualization. As other softwares, Visbrain includes graphical user interfaces for higher level interactions with visual primitives. But the greatest novelty and added-value of Visbrain lies in its structure and especially the object level which, once configured properly, can offer a great modularity for designing figures and layout that reflect brain data results. This package is also configured and tested on continuous integration servers to improve its robustness on different platforms using Travis (Linux) and AppVeyor (Windows). In addition, the documentation is built and deployed automatically using CircleCi. This also implies that Visbrain can be used on a remote server in headless mode.

### Limitations and Perspectives

Although much effort has been devoted in providing a software compatible with multi-modal data, it is not equally featured across recording techniques. For example, fMRI cross-section is still a beta feature and electrocorticographic data-specific visualization tools are not implemented so far. Secondly, efforts must now be made to make Visbrain fully compatible with Jupyter in order to have visuals embedded inside notebooks and iPython for interactive shell. We are also considering adding the compatibility with the Brain Imaging Data Structure (BIDS; Gorgolewski et al., 2016; Niso et al., 2018), a set of guidelines for organizing behavioral, MRI and M/EEG data that facilitates data sharing and reproducibility. Finally, Visbrain also contains experimental functions for the compatibility with MNE-Python (Gramfort et al., 2013), but this compatibility will be substantially enhanced in the future.

## Conclusions

In summary, Visbrain is a Python open-source and cross-platform software for brain data visualization which provides, among others, the following features: (1) GPU-powered graphical rendering providing efficient data plotting, even for large datasets and real-time interactions. (2) Modularity and flexibility with respect to users' specific needs through neuro-oriented visual primitives that can be juxtaposed or superimposed into subplots, following a Matplotlib-like behavior. (3) Complete control over the aesthetic through highly customizable configuration of color properties, allowing better use of this particularly informative dimension. Visbrain is in its early stages of development, but the present core should hopefully motivate users and programmers to contribute to the project and build a community-driven, powerful, sustainable, and full-featured open-source solution for brain data visualization.

## Author Contributions

EC is the main developer of this software. RV, CO, MJ, AP, AS, TT, DM, TL, and DA contributed in testing and in the development of this software and writing of the article. PR, AG, and KJ actively helped in the writing process.

### Conflict of Interest Statement

The authors declare that the research was conducted in the absence of any commercial or financial relationships that could be construed as a potential conflict of interest.

## Footnotes

^1^http://neuroimage.usc.edu/brainstorm/

^2^https://sccn.ucsd.edu/eeglab/

^3^http://www.fieldtriptoolbox.org/

^4^https://martinos.org/mne/stable/index.html

^5^https://pysurfer.github.io/

^6^http://nilearn.github.io/

^7^http://visbrain.org

^8^https://www.scipy.org/

^9^https://pandas.pydata.org/

^10^http://scikits.appspot.com/

^11^http://www.statsmodels.org/stable/index.html

^12^https://github.com/EtienneCmb/visbrain

^13^https://github.com/EtienneCmb/tensorpac

^14^https://pypi.org/project/visbrain/

^15^http://seaborn.pydata.org

^16^http://pyqtgraph.org/

^17^https://glumpy.github.io/

^18^https://www.qt.io/

^19^http://www.sphinx-doc.org/en/stable/

^20^http://visbrain.org

^21^http://visbrain.org/auto_examples/index.html

^22^http://visbrain.org/api.html#objects

^23^http://visbrain.org/api.html#module-visbrain.objects

^24^http://visbrain.org/signal.html

^25^https://github.com/vispy/vispy/blob/master/examples/demo/gloo/realtime_signals.py

^26^http://visbrain.org/brain.html

^27^http://visbrain.org/sleep.html

^28^http://visbrain.org/api.html
